# The effect of intensive family-centered health education on the awareness rate, diagnosis, and treatment of post-stroke depression in community families

**DOI:** 10.1186/s12875-022-01895-5

**Published:** 2022-11-12

**Authors:** Hao Wang, Shuchao Pan, Qiwu Xu, Ting Ding

**Affiliations:** 1 Department of Neurology, TongLing Municipal Hospital, 2999 Changjiang West Road, TongLing, 244000 Anhui China; 2 Department of Gerontology, Qinghai University Affiliated Hospital, Xining, 810000 Qinghai China

**Keywords:** Stroke, Post-stroke depression, Health education, Community treatment, Family care, Psychotherapy

## Abstract

**Objective:**

To investigate the effects of intensive family-centered health education on the awareness rate, diagnosis, and treatment of post-stroke depression (PSD) in community families.

**Methods:**

Elderly patients (60–90 years) from 20 community service centers affiliated with the Department of Neurology and the Medical Association of Tongling Municipal Hospital who had been diagnosed with stroke between January 2017 and June 2020 were screened using the hospital and community electronic medical record system. In this randomized cluster trial, 119 patients from 10 communities were assigned as the control group and received routine community treatment, while 126 patients from the other 10 communities were assigned as the experimental group and received routine treatment plus family-centered intensive health education. After 12 months of medical intervention, the assessment of PSD in the two groups was performed by a neurologist and a psychiatrist, both blind to the study design, using the Hamilton Rating Scale for Depression.

**Results:**

The awareness rates of the causes, clinical manifestations, treatment plan, and family care of PSD in the experimental group were 88.89, 91.30, 93.65, and 92.06%, respectively. In the control group, the awareness rates of these parameters were 72.27, 69.75, 71.43, and 65.55%, respectively, and the differences between the two groups were statistically significant (*P* < 0.05). In the experimental group, the rates of PSD diagnosis, prompt medical attendance, drug treatment compliance, and psychotherapeutic treatment compliance were 27.78, 22.22, 18.25, and 11.90%, respectively. In the control group, the rates of these parameters were 13.79, 6.03, 3.48, and 1.72%, respectively, and the differences between the two groups were statistically significant (*P* < 0.05).

**Conclusion:**

Intensive family-centered health education can improve the level of knowledge of PSD in the community, promote the timely treatment and diagnosis of PSD in patients, and improve the compliance rates of drug therapy and psychotherapy, so this is worthy of promotion.

## Introduction

With the acceleration of urbanization and the growth of the older population in China, the incidence of stroke is undergoing explosive growth [1]. Strokes are the leading cause of death and disability in middle-aged and elderly people, with an annual incidence growth rate of 8.3% [2]. Following specialized treatment, older patients in the acute stages of a stroke are usually discharged to families and communities for follow-up treatment, where they receive neurological rehabilitation exercises, drugs for secondary prevention, and further treatments [3, 4]. However, this practice often ignores the potential for the development of psychological and psychiatric disorders, such as post-stroke depression (PSD) [5]. A previous study has found that delayed diagnosis and treatment of PSD may lead to stroke recurrence and suicide [6]. This is closely related to a lack of health education in community prevention and treatment programs and a lack of PSD knowledge within community families [7]. However, few studies have reported on intensive family-centered health education in community families in China and its effects on PSD. This study, in cooperation with the local community hospital and its departments of neurology and psychiatry, aims to investigate whether an intensive health education program, compared with routine community treatment, could contribute to the early identification, diagnosis, early treatment of PSD.

## Materials and methods

### Research subjects

Senior patients (60–90 years) from 20 community service centers affiliated with the Department of Neurology and the Medical Association of Tongling Municipal Hospital who had been diagnosed with stroke (an acute cerebral infarction diagnosis within 2 weeks of its onset) between January 2017 and June 2020 were screened using the hospital and community electronic medical record systems. In this randomized cluster trial, 119 patients from 10 communities were assigned as the control group and received routine treatment, while 126 patients from the other 10 communities were assigned as the experimental group and received routine treatment plus family-centered intensive health education. After 12 months of medical intervention, the assessment of PSD in the two groups was performed by a neurologist and a psychiatrist, both blind to the study design, using the Hamilton Depression Scale (HAM-D).

The inclusion criteria were as follows: (1) Patients met the diagnostic criteria for stroke established by the 4th National Cerebrovascular Disease Academic Conference in 2018 [8], and the diagnosis was confirmed on imaging. (2) Patients were aged over 60 years. (3) Patients had clear consciousness with no cognitive or communication barriers following discharge. (4) Patients had one primary caregiver. (5) Patients volunteered to participate in the study and gave written informed consent.

The exclusion criteria were as follows: (1) Patients had heart, liver, or kidney complications or serious dysfunction in other organs. (2) Patients had a mental or psychological illness (either a previous history of mental illness or a new-onset mental illness as evaluated by the HAM-D). (3) Patients had a malignant tumor (or tumors). (4) Patients had severe hearing impairments. (5) Patients were unable to complete a follow-up visit.

The inclusion/exclusion criteria for the caregivers were as follows: (1) The primary caregivers of the patients who suffered from strokes were relatives (employers were excluded). (2) Caregivers must have been educated to a primary school level or above and have had basic reading and writing abilities. (3) Caregivers who had any serious illnesses or mental disorders were excluded. (4) Caregivers were aged between 18 and 60 years. (5) Caregivers volunteered to participate in the study and gave written informed consent.

This study was approved by the medical ethics committee of the Tongling Municipal Hospital.

### Methods

#### The control group received routine community treatment

Routine community treatment included (1) drug therapy such as anti-platelet therapy and medication for lipid regulation and plaque stabilization, (2) other treatments such as rehabilitation training for paralysis in patients with neurological impairment, and (3) providing information to the caregivers regarding routine care and life guidance.

#### The experimental group received combined intensive health education

An intensive health education program was delivered with the following: (1) Professional health education groups were set up in psychiatry departments, neurology departments, and community hospitals to strengthen health education. (2) An educational plan based on the Manual of Post-Stroke Depression was developed, and the educational content included causes of the disease, clinical manifestations, treatment, how to carry out family care, and diet and rehabilitation guidance. Each subject in the experimental group received the same lectures. (3) Special lectures were organized and delivered by PSD experts, which were held monthly for both patients and caregivers and included video presentations on PSD. (4) Neurology specialists carried out regular home visits once every two months. The home visit served to closely communicate with the patients, detect patients with suspected symptoms (psychiatric symptoms that were scored by the HAM-D), notify patients of treatment in specialized hospitals, and initiate a WeChat communication group to allow patients and caregivers to consult in real time about changes in their condition.

### Main outcome measures

#### Comparison of disease knowledge between the two groups of families

Patients and their caregivers were classified as one family response unit. The disease knowledge of the families from the two groups was investigated using tests. The content of the tests included four aspects: The causes of the disease, its clinical manifestations, treatment plans, and family care. For each family, the correct answers to each question were counted, and percentages were then calculated.

#### Comparison of the diagnosis and treatment of post-stroke depression between the two groups

The assessment of PSD was performed by a neurology specialist within one year of the onset of the stroke. The neurologist used the 24-item score of the HAM-D (< 8, normal; 8–20, mild depression; 21–35, moderate depression; and > 35, severe depression). The primary outcome indicators included the rates of diagnosis, prompt medical attendance, and compliance with drug and psychotherapeutic treatments. Diagnosis referred to patients diagnosed with PSD who had their caregivers accompany them to the psychiatric department. The prompt medical attendance referred to patients’ medical treatment behavior in the two months from the onset of symptoms to the diagnosis of PSD in the psychiatric department. The compliance with drugs referred to the patient taking an antidepressant medication as prescribed. Finally, psychotherapy compliance referred to the acceptance and adherence to adjunctive treatment provided by formal psychological medical institutions according to the medical advice of clinicians. The number of patients who met each of the above requirements was counted, and percentages were then calculated. If depression or a depressive state was diagnosed after the evaluation by the HAM-D, antidepressant treatment was applied.

### Statistical method

The Student’s *t*-test was used to analyze the measurement data, and the chi-squared test was used to analyze the enumeration data. A *P* value of < 0.05 was considered statistically significant. Analyses were carried out using the SPSS software version 19.0.

## Results

### Comparison of socio-demographic and clinical characteristics between the two groups

In the control group, there were 69 men and 50 women, and their average age was 70.68 ± 6.40 years. Eighty-three patients had completed junior high school, and thirty-six had completed elderly high school. The sites of cerebral infarction in the patients were as follows: The basal ganglia (*n* = 85), the frontal lobe (*n* = 9), the temporal lobe (*n* = 6), the parietal lobe (*n* = 3), and multiple cerebral infarction locations (*n* = 16).

In the experimental group, there were 73 men and 53 women, and their average age was 71.26 ± 6.67 years. Eighty-seven patients had completed junior high school, and thirty-nine had completed elderly high school. The sites of cerebral infarction in the patients were as follows: The basal ganglia (*n* = 87), the frontal lobe (*n* = 10), the temporal lobe (*n* = 7), the parietal lobe (*n* = 5), and multiple cerebral infarction locations (*n* = 17). There were no statistically significant differences in these parameters between the two groups (*P* > 0.05).

In the control group, the caregiver relationships with the patients were as follows: 61 were spouses, 48 were children, and 10 were siblings. In the experimental group, the caregiver relationships with the patients were as follows: 64 were spouses, 51 were children, and 11 were siblings. There were no statistically significant differences in caregiver–patient relationships between the two groups (*P* > 0.05).

### Comparison of disease knowledge between the two groups of families

The awareness rate of the causes of PSD was 88.89% in the experimental group and 72.27% in the control group, which was statistically significant (*P* < 0.05). The awareness rate of the clinical manifestations of PSD was 91.30% in the experimental group and 69.75% in the control group, which was statistically significant (*P* < 0.05). The awareness rates of treatment plans and family care for PSD were 93.65 and 92.06%, respectively, in the experimental group and 71.43 and 65.55%, respectively, in the control group, which were statistically significant (both *P* < 0.05). These figures are illustrated in Table 1.

**Table 1:** Comparison of disease knowledge between the two groups of families [case(%)]

Group	n	Awareness rates of the causes	Clinical manifestations	Treatment plan	Family care of PSD
Control group	119	86(72.27%)	83(69.75%)	85(71.43%)	78(65.55%)
Experimental group	126	112(88.89%)	115(91.30%)	118(93.65%)	116(92.06%)
χ2	–	10.90	18.29	21.28	26.11
*P* value	–	< 0.01	< 0.01	< 0.01	< 0.01

### Comparison of the diagnosis and treatment of post-stroke depression between the two groups

Patients with a frontotemporal brain injury or a larger injury area were more likely to have a higher HAM-D score. Those who initially scored over 20 showed significant improvement (*P* < 0.05), while those who initially scored under 20 showed no significant improvement (*P* > 0.05). After 12 months of intervention, the HAM-D score of the experimental group was significantly lower than that of the control group.

The disease diagnosis rate was 27.78% in the experimental group and 13.79% in the control group. The rate of prompt medical attendance was 22.22% in the experimental group and 6.03% in the control group. The drug and psychotherapeutic treatment compliance rates were 18.25 and 11.90%, respectively, in the experimental group and 3.48 and 1.72%, respectively, in the control group (see Table 2). Taken together, the compliance rates of medical and psychotherapeutic treatments in the experimental group were significantly higher than in the control group. The symptom improvement rate of patients in the experimental group was also significantly higher than that in the control group.

**Table 2:** Comparison of the diagnosis and treatment of PSD between the two groups [case(%)]

Group	n	Rates of disease diagnosis	Timely attendance	Drug treatment compliance	Psychological treatment compliance
Control group	119	16(13.79%)	7(6.03%)	4(3.48%)	2(1.72%)
Experimental group	126	35(27.78%)	28(22.22%)	23(18.25%)	15(11.90%)
χ2	–	7.63	13.34	13.84	9.91
*P* value	–	< 0.01	< 0.01	< 0.01	< 0.01

## Discussion

Post-stroke depression is a type of mental illness secondary to stroke. Patients who have suffered strokes and then developed PSD have been reported to have higher rates of mortality than patients who have suffered strokes that did not develop PSD [9, 10]. The present study showed that intensive family-centered health education could improve the level of knowledge of PSD in the community and promote the timely treatment and diagnosis of PSD patients.

The incidence rate of PSD is time-dependent. A high incidence of PSD occurs from weeks to months following the onset of stroke [11]. A study showed that the incidence of PSD within two weeks of a stroke was 28.1%, one year following a stroke, the incidence had risen to 61%, and, at two years, it had reached 79% [12]. Compared with men, women are significantly less likely to suffer from depression within 90 days of a stroke and more likely to accept drug treatment for PSD [13]. The incidence of stroke complications is as high as 33.5% [14]. Clinical manifestations can include low spirits, retardation of thinking, sleep disorders, slow reaction, anxiety, world-weariness, and hallucinations [15, 16]. In severe cases, suicidal ideation may be present, which can seriously affect the recovery of neurological function and quality of life. Moreover, PSD increases stroke recurrence rate, further aggravating disability and daily living ability [17]. The pathogenesis of PSD has not been clarified either at home or abroad [18], so early identification, diagnosis, and treatment are particularly important. It is highly important for the families of patients in the community who have suffered from a stroke to identify PSD early so that a timely diagnosis can be made, allowing treatment interventions that can impact the disease’s development.

Psychiatry and neurology departments should also cooperate with community doctors to provide health education for families of patients who have suffered from strokes. This education includes popularizing the etiologies, clinical manifestations, diagnoses, treatment methods, family care, and other related information on PSD. Delivering this knowledge involves holding lectures, using modern information technology and equipment to prepare and regularly distribute learning videos and complementary animations, and organizing professional teams to guide teaching and provide disease consultation [19].

The results of this study have shown that the rate of PSD knowledge in the experimental group was higher than that in the control group, and this included the etiologies, clinical manifestations, diagnoses, and treatments for this condition. Strengthening health education can effectively eliminate patient concerns, change their concept of medical treatment, improve family care, and ensure the accurate judgment of the clinical manifestations of diseases in a short time frame. In addition, prompt visits to a psychiatric department improve adherence and compliance with drug therapy and psychotherapy.

This study had some limitations. First, the sample size was relatively small, and all subjects were from the community centers in one area, although as many eligible patients as possible were recruited from the medical record system. It probably explains why the PSD diagnosis rate in the experimental group was twice as high as in the control group. Patients with a history of depression were not included, and all PSD patients could not be fully covered, resulting in incomplete research data. Second, this study did not examine whether PSD would aggravate the disability and daily living ability of patients. Third, the duration of intensive family-centered health education was relatively short. Future studies are needed to investigate how to design more effective interventions to improve the mental health of patients after stroke [20]. In addition, the tests used to assess disease knowledge were developed by the study team and had not been validated. Fourth, as the clinical data of patients were retrospectively reviewed, the characteristics of comorbidities and stroke severity/outcomes were not available for all patients. Finally, provider factors may also play a role in delayed diagnosis and treatment of PSD, which warrants further investigation.

## Conclusion

In conclusion, although intensive family-centered health education was time-limited, it effectively improved the knowledge and understanding of PSD within community families. This can be considered an important task for community-related chronic disease management education, which improves the early diagnosis and treatment of diseases and reduces the burden of diseases on families and society.

## Data Availability

The datasets used and/or analysed during the current study available from the corresponding author on reasonable request.
